# Prothrombin Complex Concentrate vs Conservative Management in ICH Associated With Direct Oral Anticoagulants

**DOI:** 10.1001/jamanetworkopen.2023.54916

**Published:** 2024-02-06

**Authors:** Bonaventure Ip, Sangqi Pan, Zhong Yuan, Trista Hung, Ho Ko, Xinyi Leng, Yuying Liu, Shuang Li, Sing Yau Lee, Cyrus Cheng, Howard Chan, Vincent Mok, Yannie Soo, Xiaoli Wu, Leong Ting Lui, Rosa Chan, Jill Abrigo, Qi Dou, David Seiffge, Thomas Leung

**Affiliations:** 1 Department of Medicine and Therapeutics, Faculty of Medicine, The Prince of Wales Hospital, The Chinese University of Hong Kong, Hong Kong SAR; 2 Li Ka Shing Institute of Health Sciences, Faculty of Medicine, The Chinese University of Hong Kong, Hong Kong SAR; 3 Department of Computer Science and Engineering, The Chinese University of Hong Kong, Hong Kong SAR; 4 Department of Electrical Engineering, The City University of Hong Kong, Hong Kong SAR; 5 Department of Diagnostic Imaging and Interventional Radiology, The Chinese University of Hong Kong, Hong Kong SAR; 6 Department of Neurology, Inselspital University Hospital Bern and University of Bern, Bern, Switzerland

## Abstract

**Importance:**

Intracerebral hemorrhage (ICH) associated with direct oral anticoagulant (DOAC) use carries extremely high morbidity and mortality. The clinical effectiveness of hemostatic therapy is unclear.

**Objective:**

To compare the clinical and radiological outcomes of DOAC-associated ICH treated with prothrombin complex concentrate (PCC) vs conservative management.

**Design, Setting, and Participants:**

In this population-based, propensity score–weighted retrospective cohort study, patients who developed DOAC-associated ICH from January 1, 2016, to December 31, 2021, in Hong Kong were identified. The outcomes of patients who received 25 to 50 IU/kg PCC with those who received no hemostatic agents were compared. Data were analyzed from May 1, 2022, to June 30, 2023.

**Main Outcomes and Measures:**

The primary outcome was modified Rankin scale of 0 to 3 or returning to baseline functional status at 3 months. Secondary outcomes were mortality at 90 days, in-hospital mortality, and hematoma expansion. Weighted logistic regression was performed to evaluate the association of PCC with study outcomes. In unweighted logistic regression models, factors associated with good neurological outcome and hematoma expansion in DOAC-associated ICH were identified.

**Results:**

A total of 232 patients with DOAC-associated ICH, with a mean (SD) age of 77.2 (9.3) years and 101 (44%) female patients, were included. Among these, 116 (50%) received conservative treatment and 102 (44%) received PCC. Overall, 74 patients (31%) patients had good neurological recovery and 92 (39%) died within 90 days. Median (IQR) baseline hematoma volume was 21.7 mL (3.6-66.1 mL). Compared with conservative management, PCC was not associated with improved neurological recovery (adjusted odds ratio [aOR], 0.62; 95% CI, 0.33-1.16; *P* = .14), mortality at 90 days (aOR, 1.03; 95% CI, 0.70-1.53; *P* = .88), in-hospital mortality (aOR, 1.11; 95% CI, 0.69-1.79; *P* = .66), or reduced hematoma expansion (aOR, 0.94; 95% CI, 0.38-2.31; *P* = .90). Higher baseline hematoma volume, lower Glasgow coma scale, and intraventricular hemorrhage were associated with lower odds of good neurological outcome but not hematoma expansion.

**Conclusions and Relevance:**

In this cohort study, Chinese patients with DOAC-associated ICH had large baseline hematoma volumes and high rates of mortality and functional disability. PCC treatment was not associated with improved functional outcome, hematoma expansion, or mortality. Further studies on novel hemostatic agents as well as neurosurgical and adjunctive medical therapies are needed to identify the best management algorithm for DOAC-associated ICH.

## Key Points

**Question:**

Is prothrombin complex concentrate (PCC) treatment useful for patients who develop intracerebral hemorrhage (ICH) during direct oral anticoagulant (DOAC) usage?

**Findings:**

In this cohort study of 232 patients in Hong Kong, 31% of patients with DOAC-associated ICH achieved good neurological recovery and 39% died within 90 days. PCC treatment was not associated with neurological outcomes, hematoma expansion, or survival.

**Meaning:**

In this study, PCC treatment was not associated with improved functional outcome, hematoma expansion, or mortality among patients with DOAC-associated ICH.

## Teaser

This cohort study compares the clinical and radiological outcomes of direct oral anticoagulant (DOAC)–associated intracerebral hemorrhage (ICH) treated with prothrombin complex concentrate vs conservative management among patients in Hong Kong.

## Introduction

Intracerebral hemorrhage (ICH) is the most severe complication during direct oral anticoagulant (DOAC) usage,^1^ with ethnicity, hypertension, advanced age, and cerebral small vessel disease being important risk factors of the condition.^2,3^ Although DOAC-associated ICH has extremely high mortality and morbidity,^4,5^ clinical effectiveness of current hemostatic strategies is unclear.^4,5,6^

Hemostasis may be achieved through (1) specific DOAC reversal agents, such as idarucizumab for dabigatran and andexanet alpha for factor Xa inhibitors,^7,8^ or (2) unspecific repletion with prothrombin complex concentrate (PCC).^5^ To date, PCC is the main hemostatic agent used for DOAC-associated ICH worldwide, as specific DOAC reversal agents, especially andexanet alpha, are costly and of limited availability.^9^ Although ascertaining the clinical benefit of PCC is crucial in devising an effective hemostatic algorithm for DOAC-associated ICH, published studies are scarce. Two small-scale observational studies from the United States^10,11^ showed conflicting results: one study^10^ suggested comparable rates of hemostasis between the 4-factor PCC and andexanet alfa in the ICH subgroup. Yet, in the other study,^11^ good clinical outcomes occurred significantly less frequently in patients treated with 4-factor PCC compared with those with andexanet alpha. Two other European studies^12,13^ suggested that PCC may not reduce hematoma expansion compared to conservative management in cohorts mainly of rivaroxaban users. The inconsistent results could be due to confounding effects of non-ICH hemorrhages, concurrent use of antiplatelet agents, and predominate use of a single DOAC. In this population-based cohort study, we aimed to compare the functional and radiological outcomes of PCC treatment in Chinese patients with DOAC-associated ICH vs conservative management.

## Methods

### Study Design and Data Source

We performed a population-based retrospective cohort study and identified all Chinese patients who developed DOAC-associated ICH admitted to public hospitals in Hong Kong from January 1, 2016, to December 31, 2021. We retrieved all clinical data via the Hospital Authority Data Collaboration Laboratory (HADCL) from January 1, 2016, to December 31, 2019. HADCL is an infrastructure under the statutory Hospital Authority that stores clinical, diagnostic, prescription, laboratory, and radiological data of all public hospitals which provide inpatient care for more than 90% of the 7.5 million residents of Hong Kong.^14,15^ We retrieved the same set of data from a territory-wide DOAC registry from January 1, 2020, to December 31, 2021, which recorded all patients who developed DOAC-associated ICH admitted to the same public hospitals in Hong Kong as those included in the HADCL. We followed the Strengthening the Reporting of Observational Studies in Epidemiology (STROBE) reporting guidelines. The Joint CUNK-NTEC Clinical Research Ethics Committee approved the study. The need for written informed consent was waived due to the use of deidentified data.

### Data Collection

We identified Chinese patients who developed DOAC-associated ICH while taking apixaban, dabigatran, edoxaban, or rivaroxaban during the study period. Clinical data (sex, age, smoking and drinking history, death, admission systolic and diastolic blood pressure, Glasgow Coma Scale [GCS], modified Rankin scale [mRS] before and 90 days after DOAC-associated ICH, and last-known-well [LKW]–to-imaging time) and laboratory information (creatinine level, alanine transferase level, platelet level, hemoglobin level, activated partial thromboplastin time [APTT] and prothrombin time [PT]) were collected. Based on *International Classification of Diseases, Ninth Revision, Clinical Modification *(*ICD-9-CM*), we retrieved the diagnoses of atrial fibrillation, ischemic stroke, intracranial hemorrhage, congestive heart failure, hypertension, diabetes, ischemic heart disease, and venous thromboembolism (eTable 1 in Supplement 1). We recorded the type and dosages of DOAC and the use of antiplatelet agents (clopidogrel, aspirin, cilostazol, ticagrelor), statins, antiseizure medications and antihypertensive medications immediately before the episode of DOAC-associated ICH. Neurosurgical procedures, including external ventricular drainage, craniotomy, craniectomy, burr hole drainage, and clot evacuation, were documented. By reviewing prescription records, consultation summaries, radiology reports, and computed tomography (CT) films, we excluded patients with last DOAC intake more than 24 hours before presentation, valvular replacement, subdural hematoma (SDH), epidural hematoma (EDH), subarachnoid hemorrhage (SAH), and intraventricular hemorrhage (IVH) without the presence of intraparenchymal hematoma. Patients with ICH secondary to hemorrhagic infarct; traumatic brain injury; cerebral venous thrombosis; vascular anomalies, such as intracranial aneurysms or arteriovenous malformations; and thrombolytic therapy were also excluded.

### DOAC-Associated ICH and Hematoma Expansion

We classified DOAC-associated ICH according to its location into lobar, basal ganglion, thalamic, and infratentorial ICH. Any concurrent IVH, SDH, EDH, SAH, and mass effect (hydrocephalus, midline shift of more than 5 mm or brain herniation) were documented. We identified ICH from noncontrast brain CT scans with slice thickness ranging from 4.98 to 5.34 mm and pixel spacing ranging from 0.429 to 0.488 mm. ICH volumes were computed by an nnU-net–based algorithm, a state-of-the-art deep learning–based method specialized for biomedical image segmentation.^16^ Dice similarity score of the algorithm for intraparenchymal hemorrhage reached a mean (SD) of 0.83 (0.12) during internal validation. Examples of the segmentation images are illustrated in the eFigure in Supplement 1. Hematoma expansion was defined as an increase in hematoma volume of more than 33% or an absolute increase by more than 6 mL compared with the baseline imaging for patients who received a sequential brain CT scan within 3 to 72 hours after the initial imaging.^13,17^ All films and segmentations were reviewed by a neurologist (B.I. or T.L.) or neuroradiologist (J.A.) blinded from the type of hemostatic treatment. Disparities with algorithmic segmentation were resolved by manual correction.

### Hemostatic Therapy for DOAC-Associated ICH

Hemostatic therapies were categorized into 3 groups. First, conservative management was defined as patients who did not receive any DOAC reversal agents. These patients received close monitoring of neurological status and vital signs in the medical or neurosurgical unit, with a systolic blood pressure target of less than 140 mm Hg. Second, the PCC group was defined as patients who received 4-factor PCC for the ICH episode in addition to close neurological monitoring and blood pressure control. A protocol-driven dosage of 25 to 50 IU/kg PCC was used to treat DOAC-associated major bleeding during the study period. The total dose of PCC given within 24 hours of emergency admission was retrieved. Third, the idarucizumab group was defined as patients who received 5 g of idarucizumab for the ICH episode. Andexanet alfa was not included, as the drug was not available in Hong Kong. We excluded patients who received both PCC and idarucizumab for the same ICH episode as well as those who received recombinant factor VII, fresh frozen plasma, or platelet concentrate.

### Outcome Definitions

The primary outcome was good neurological recovery, defined as an mRS score of 0 to 3, or returning to baseline mRS after DOAC-associated ICH at 90 days. Secondary outcomes were all-cause mortality at 90 days, in-hospital mortality, and hematoma expansion among patients who received progress CT imaging of the brain 3 to 72 hours after the initial scan.

### Statistical Analysis

We developed a propensity score (PS)–weighting model for comparisons between the average treatment effect of PCC treatment and conservative management by inverse probability of treatment weighting to minimize the effects of confounding variables. The PS of each patient was estimated by generalized boosted models (GBM) that involved covariates including age, sex, baseline mRS, concurrent antiplatelet therapy, blood pressure, GCS, initial hematoma volume, hematoma location, IVH, platelet count, APTT, and neurosurgical procedures as covariates. The optimal iteration of GBM was determined by 4 stopping rules described previously^18^; the stopping rule with the best covariate balance and effective sample size was selected. Covariate balance between the PCC and conservative treatment groups was assessed by the absolute standardized mean difference (ASMD), defined as the difference in means of the covariate divided by the pooled SD of both groups.^19^ Covariates that failed to achieve an ASMD of less than 0.2 and other imbalanced clinical parameters were adjusted in a doubly robust model. In the primary analysis, we compared neurological recovery at 90 days and hematoma expansion with respect to hemostatic therapy in a weighted logistic regression model. Weighted Cox regression was used to analyze all-cause death after assessing the proportional hazard assumptions by log-log plot of survival. We used the Kaplan-Meier method to estimate the cumulative incidence of death. The same analysis was performed for the atrial fibrillation subgroup.

We conducted 2 unweighted analyses to identify factors associated with good neurological recovery and hematoma expansion by multivariate logistic regression for a priori covariates of interest based on current literature,^1,17,20^ including (1) systolic blood pressure of less than 160 mm Hg, (2) LKW-to-CT time, (3) location of ICH (infratentorial vs noninfratentorial), (4) baseline hematoma volume, (5) antiplatelet therapy, (6) IVH, and (7) admission GCS. We also performed unweighted multivariate logistic regression in a data-driven approach as a sensitivity analysis for factors associated with good neurological recovery. Covariates that reached a *P* ≤ .10 in the univariate logistic regression analyses and variables that were independently associated with neurological recovery in the a priori model were subjected to a multivariate logistic regression model. Due to the prolonged study period (January 1, 2016, to December 31, 2021), we performed a temporal analysis that dichotomized patients by the median date of DOAC-associated ICH onset to evaluate any time-dependent effects on study outcomes.

Assuming missing baseline data to be missing at random, we substituted missing data with values computed by multiple imputation with chained equations that created 20 complete datasets after the first 20 iterations. Missing data on glycated hemoglobin A_1c_ (29%), LKW-to-CT time (18%), alanine aminotransferase level (1%), creatinine level (1%), premorbid mRS (1%), 3-month mRS (1%), and admission GCS (0.4%) were imputed and kept within reasonable ranges. Normally distributed continuous variables, nonnormally distributed continuous variables, and categorical variables were expressed as mean with SD, median with IQR, and number with percentage, respectively. Baseline characteristics were compared by independent sample *t* test or weighted Mann-Whitney *U* test for continuous variables, and weighted χ^2^ test or Fisher exact test were used for categorical variables. A 2-sided test with *P* < .05 was considered statistically significant. All statistical analyses were performed with RStudio version 1.4.1717 (R Project for Statistical Computing).

## Results

We identified 232 patients with DOAC-associated ICH from January 1, 2016, to December 31, 2021. Mean (SD) age was 77.2 (9.3) years, and 101 (44%) were female patients. In terms of hemostatic therapy, 116 (50%) received conservative treatment, 102 (44.0%) received PCC (median [IQR] dosage, 2000 IU [1500-2500 IU]), and 14 (6.0%) received idarucizumab (all received a dosage of 5 g) (Figure). Idarucizumab was excluded from the primary analysis due to the small sample size. There were 117 patients (50%) receiving rivaroxaban; 62 (27%), apixaban; 48 (21%), dabigatran; and 5 (2%), edoxaban. Median (IQR) duration of DOAC use was 18 (7-35) months. Median (IQR) LKW-to-CT time was 3.9 (1.3-10.1) hours. Median (IQR) initial hematoma volume was 21.7 (3.6-66.1) mL. Overall, 74 patients (31%) achieved good neurological recovery, and 92 (39%) died within 90 days. Sequential CT head scans were available for 130 patients (56.0%) within 3 to 72 hours after the initial imaging, among whom 40 (31%) developed hematoma expansion. All covariate balance in the propensity score–weighted model reached an ASMD of less than 0.2. Table 1 summarizes the characteristics of the weighted cohort. eTable 2 in Supplement 1 summarizes the characteristics of the unweighted cohort.

**Figure.**
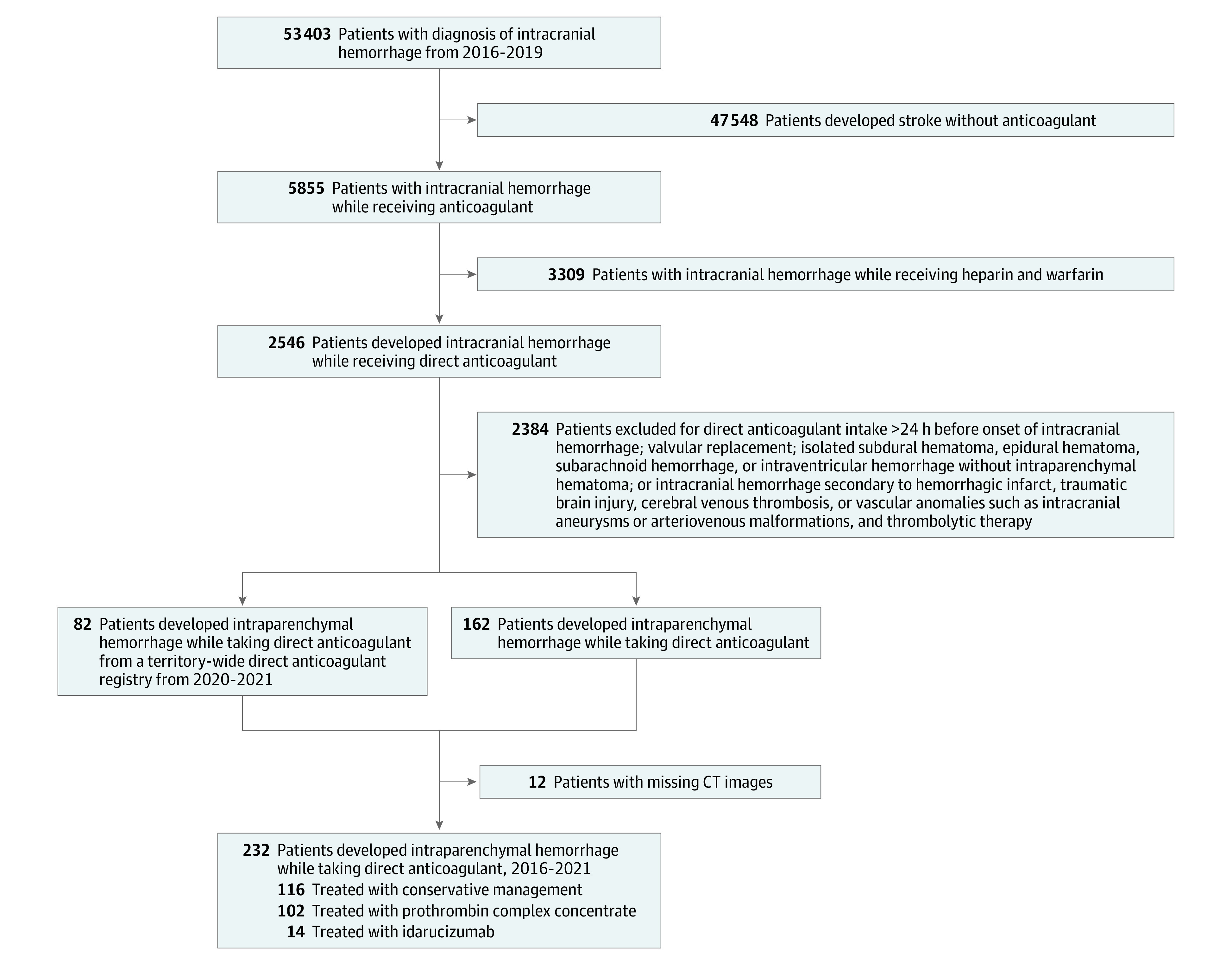
Study Flow Diagram CT indicates computed tomography.

**Table 1.** Characteristics of the Weighted Cohort With DOAC-Associated ICH

Characteristic	Patients, No. (%)		*P* value
	PCC (n = 85)	Conservative (n = 97)	
DOAC dosage, median (IQR), mg			
Apixaban	5.00 (5.00-10.00)	5.00 (5.00-10.00)	.84
Dabigatran	220.00 (220.00-300.00)	220.00 (220.00-220.00)	.17
Edoxaban	30.00 (30.00-60.00)	30.00 (30.00-60.00)	.71
Rivaroxaban	20.00 (15.00-20.00)	20.00 (15.00-20.00)	.74
Demographic characteristics			
Age, mean (D), y	76.71 (9.17)	76.58 (9.58)	.92
Sex			
Female	39 (46)	46 (47)	.90
Male	46 (54)	51 (53)	
Ever smoker	26 (30)	31 (32)	.76
Ever drinker	20 (23)	27 (28)	.46
Medical comorbidities			
Atrial fibrillation	75 (88)	79 (81)	.24
Congestive heart failure	13 (15)	8 (8)	.13
Hypertension	63 (74)	66 (68)	.34
Diabetes	30 (35)	29 (30)	.50
Ischemic stroke	12 (14)	16 (16)	.72
Ischemic heart disease	19 (22)	23 (24)	.68
Major bleeding	9 (10)	2 (2)	.06
Venous thromboembolism	5 (6)	13 (13)	.19
History of ICH	0	0	NA
Epilepsy	6 (7)	10 (10)	.52
Laboratory tests, median (IQR)			
APTT, s	35.20 (30.20-38.30)	34.70 (28.50-37.30)	.07
PT, s	13.80 (12.10-16.40)	13.80 (12.40-16.50)	.51
Alanine aminotransferase, IU/L	19.00 (16.00-27.00)	18.00 (13.60-27.00)	.18
Creatinine, μmol/L	90.00 (72.00-112.00)	94.00 (74.00-117.00)	.26
Hemoglobin, g/dL	13.30 (11.90-14.30)	13.00 (11.60-14.10)	.23
Platelet, ×10^3^/μL	207.00 (150.00-248.00)	195.00 (157.00-267.00)	.79
HbA_1c_, %	6.00 (5.70-6.60)	6.00 (5.70-6.60)	.96
Concurrent medications			
Antiplatelet	16 (19)	16 (17)	.77
Antiseizure medication	9 (11)	4 (4)	.06
Nitrate	18 (21)	32 (33)	.08
Calcium channel blocker	48 (57)	74 (76)	.005
β-Blocker	64 (75)	76 (78)	.56
Statin	54 (63)	67 (69)	.41
ICH location			
Basal ganglia	30 (35)	27 (28)	.35
Thalamus	20 (23)	20 (21)	.73
Lobar	37 (43)	40 (41)	.81
Cerebellum or brainstem	18 (21)	20 (21)	>.99
Concurrent intracranial abnormalities			
Subdural hemorrhage	4 (5)	5 (5)	>.99
Intraventricular hemorrhage	22 (26)	27 (28)	.84
Subarachnoid hemorrhage	6 (7)	6 (6)	.84
Midline shift, hydrocephalus, or herniation	34 (40)	39 (40)	.97
Clinical and radiological parameters on admission			
Systolic blood pressure, mean (SD), mm Hg	175.33 (27.19)	175.00 (24.50)	.93
Diastolic blood pressure, mean (SD), mm Hg	104.40 (17.24)	103.78 (18.83)	.82
Admission Glasgow Coma Scale, median (IQR)	14.00 (9.00-15.00)	14.00 (9.00-15.00)	.76
Premorbid modified Rankin scale, median (IQR)	2.00 (0-3.00)	1.00 (0-3.00)	.83
LKW-to-CT time, median (IQR), h	3.4 (1.3-13.1)	3.9 (1.5-15.9)	.22
Baseline hematoma volume, median (IQR), mL	17.61 (4.19-52.18)	24.53 (2.60-61.12)	.94
Neurosurgical procedures	5 (6)	6 (6)	.88
Outcomes			
Good neurological outcome	22 (26)	35 (36)	.13
Mortality at 90 d	35 (41)	37 (38)	.60
In-hospital mortality	33 (39)	37 (38)	.86

Abbreviations: APTT, activated partial thromboplastin time; CT, computer tomography; DOAC, direct oral anticoagulant; HbA_1c_, hemoglobin A_1c_; ICH, intracerebral hemorrhage; LKW, last-known-well; NA, not applicable; PCC, prothrombin complex concentrate; PT, prothrombin time.

SI conversion factors: To convert alanine aminotransferase to microkatals per liter, multiply by 0.0167; creatinine to milligrams per deciliter, multiply by 0.0113; HbA_1c_ to proportion of total hemoglobin, multiply by 0.01; hemoglobin to millimoles per liter, multiply values by 0.6206; and platelets to cells ×10^9^ per liter, multiply by 1.

### Primary Analysis

PCC treatment was not associated with good neurological recovery (adjusted odds ratio [aOR], 0.62; 95% CI, 0.33-1.16; *P* = .14), mortality at 90 days (aOR, 1.03; 95% CI, 0.70-1.53; *P* = .88) or in-hospital mortality (aOR, 1.11; 95% CI, 0.69-1.79; *P* = .66) compared with conservative management (Table 2). Among the 96 patients with sequential CT head scan in the weighted cohort (eTables 3 and 4 in Supplement 1), PCC treatment was not associated with good neurological recovery (aOR, 0.50; 95% CI, 0.21-1.24; *P* = .14), mortality at 90 days (aOR, 1.24; 95% CI, 0.64-2.43; *P* = .52), in-hospital mortality (aOR, 1.37; 95% CI, 0.59-3.20; *P* = .46), or reduced hematoma expansion (aOR, 0.94; 95% CI, 0.38-2.31; *P* = .90). In the atrial fibrillation subgroup, PCC treatment was also not associated with primary and secondary outcomes (eTable 5 and 6 in Supplement 1).

**Table 2.** Weighted Regression of Study Outcomes

Outcomes	Prothrombin complex concentrate (n = 85) vs conservative management (referent, n = 97)	
	aOR (95% CI)	*P* value
Good neurological recovery at 90 d	0.62 (0.33-1.16)	.14
Mortality at 90 d	1.03 (0.70-1.53)	.88
In-hospital mortality	1.11 (0.69-1.79)	.66
Hematoma expansion^a^	0.94 (0.38-2.31)	.90

Abbreviation: aOR, adjusted odds ratio.

^a^ Overall, 48 patients in the prothrombin complex concentrate group and 48 patients in the conservative management group had sequential brain computed tomography images for comparison of hematoma expansion.

### Secondary Analyses

#### Factors Associated With Good Neurological Outcome

In the multivariate logistic regression model for a priori covariates, higher baseline hematoma volume (aOR, 0.97; 95% CI, 0.96-0.99; *P* = .002) and IVH (aOR, 0.24; 95% CI, 0.08-0.71; *P* = .01) were associated with lower odds of good neurological recovery. A higher GCS on admission (aOR, 1.27; 95% CI, 1.09-1.47; *P* = .002) was associated with higher likelihood of good neurological recovery (Table 3).

**Table 3.** Unweighted Logistic Regression With A Priori Covariates

Covariate	Multivariate model, aOR (95% CI)	*P* value
Good neurological outcome		
Systolic blood pressure <160 mm Hg	0.99 (0.98-1.01)	.51
Infratentorial intracerebral hemorrhage	1.27 (0.54-2.97)	.58
Baseline hematoma volume	0.97 (0.96-0.99)	.002
Concurrent antiplatelet agent	0.62 (0.24-1.60)	.32
Intraventricular hemorrhage	0.24 (0.08-0.71)	.01
Glasgow Coma Scale on admission	1.27 (1.09-1.47)	.002
LKW-to-CT time	1.02 (0.96-1.09)	.43
Hematoma expansion		
Systolic blood pressure <160 mm Hg	1.00 (0.98-1.02)	.96
Infratentorial intracerebral hemorrhage	1.14 (0.45-2.84)	.78
Baseline hematoma volume	1.00 (0.99-1.00)	.46
Concurrent antiplatelet agent	1.93 (0.74-5.03)	.18
Intraventricular hemorrhage	1.45 (0.58-3.67)	.43
Glasgow Coma Scale on admission	0.89 (0.78-1.01)	.06
LKW-to-CT time	0.94 (0.92-1.02)	.23

Abbreviations: aOR, adjusted odds ratio; CT, computer tomography; LKW, last-known-well.

#### Factors Associated With Hematoma Expansion

Among the 130 patients who received a second CT head scan within 3 to 72 hours after the initial scan, an a priori set of covariates, including systolic blood pressure of less than 160 mm Hg, hematoma location, LKW-to-CT time, concurrent use of antiplatelet agent, baseline hematoma volume, and IVH, were tested. None were associated with hematoma expansion (Table 3).

#### Sensitivity Analyses

In a data-driven approach, we determined the association between good neurological recovery and the covariates listed in Table 1 by univariate logistic regression (eTable 7 in Supplement 1). After subjecting covariates that reached a *P* ≤.10 (SAH, premorbid mRS, congestive heart failure, mass effect, and PCC treatment) and factors associated with good neurological outcome in the a priori model, baseline hematoma volume (aOR, 0.97; 95% CI, 0.95-0.99; *P* = .001), IVH (aOR, 0.29; 95% CI, 0.09-0.88; *P* = .03), and admission GCS (aOR, 1.21; 95% CI, 1.04-1.41; *P* = .02) remained independently associated with neurological outcome (eTable 8 in Supplement 1). Temporal analysis found no statistical differences in outcomes between earlier vs later study period (eTable 9 in Supplement 1).

## Discussion

In this population-based study of Chinese patients with DOAC-associated ICH, we found that (1) DOAC-associated ICH carried high risk of mortality and functional disability; (2) PCC treatment was not associated with improved neurological recovery, mortality at 90 days, in-hospital mortality, or reduced hematoma expansion; and (3) higher baseline hematoma volume, lower GCS on admission, and IVH were associated with poor neurological recovery. Prior to our research, there was 1 Japanese clinical study^21^ that reported a 17% in-hospital mortality of DOAC-associated ICH with a median hospital stay of 18 days, while 2 additional Japanese neuroradiological studies^22,23^ on DOAC-associated ICH had limited sample sizes (≤6 participants) with no patients receiving hemostatic therapies. Our study finding of 39% 3-month mortality depicted the longer-term trajectory of Asian patients with DOAC-associated ICH. Importantly, the death rate was slightly higher than that reported by European studies (28%-33%).^1,12,13,17,24^ The disparity may be explained by the baseline hematoma volume of our cohort, with a median value of 21.7 mL and a mean value of 48.4 mL, which was larger than that patients in of the European studies.^6,12,13,17,25^ Apart from the difference in hematoma quantification methods, potential reasons that explain the discrepancy include the prevalence of cerebral small vessel disease among Asians,^26,27^ high systolic blood pressure on presentation, and interethnic difference in DOAC metabolism.^28^ Future studies should determine whether Asian race is an independent risk factor of more severe DOAC-associated ICH.

Contrary to warfarin-related ICH,^29^ the effectiveness of PCC treatment in DOAC-associated ICH was unclear despite being a treatment option in international guidelines.^5^ Unfortunately, PCC treatment did not appear to improve functional or radiological outcomes despite robust animal data,^30,31^ possibly due to the fundamental differences in the ICH mechanisms and the heterogeneity in onset-to-treatment time, blood pressure, and medical comorbidities. Thus far, only 1 German study^12^ suggested that PCC may not be efficacious in a cohort with more than 75% rivaroxaban users, of whom only half received more than 25 IU/kg of PCC. Our study differed from the previous research in that we had a larger sample size with other DOAC users, all patients received a PCC dosage of 25 to 50 IU/kg, and it was the first study of which we are aware that evaluated the outcomes of PCC treatment among Chinese patients with DOAC-associated ICH. Our results call for timely research in alternative hemostatic therapies for DOAC-associated ICH, especially among Asian patients due to their underrepresentation in the literature, even in a recent trial that evaluated andexanet alfa for factor Xa inhibitor–related ICH with positive preliminary results (NCT03661528). Moreover, with the emergence of medical and neurosurgical management, such as the intensive medical care bundle and minimally invasive hematoma removal,^32,33^ further studies should investigate whether these treatments may benefit patients with DOAC-associated ICH in conjunction with hemostatic agents.

Lastly, similar to ICH among non-DOAC users, larger hematoma volumes, lower GCS, and IVH were associated with worse outcomes of DOAC-associated ICH.^20^ Future research should evaluate whether patients with these characteristics may benefit from early and aggressive hemostatic therapy, blood pressure lowering, neurosurgical procedures, and close neuro-intensive monitoring.^34^

### Limitations

Our study has some limitations. First, due to the observational nature of our study, we were unable to assess causality. Despite PS weighting with good covariate balance, unmeasured confounding factors may be present. For instance, early palliation may have reduced the intensity of medical management and frequency of investigations, which could have impacted outcomes and excluded patients with poor prognostic factors from hematoma expansion evaluation. This is supported by the high GCS and lower rates of adverse outcomes observed in patients who underwent sequential CT head scans. Second, despite being one of the largest cohorts of DOAC-associated ICH patients, the sample size of the current study still limited the number of covariates in the multivariate logistic regression models. Third, the exact time points of blood pressure measurement were not available. Therefore, it would be difficult to gauge the effect of blood pressure control on outcomes of DOAC-associated ICH. Fourth, as only 14 patients received idarucizumab for severe dabigatran-related ICH with a median baseline hematoma volume of 85.8 mL, statistically appropriate comparisons with other treatment groups cannot be made. Fifth, alternative hemostatic agents, such as tranexamic acid, were not evaluated. Sixth, DOAC exposure was based on prescription record and confirmation in clinical notes. Specific coagulation tests, such as anti–factor Xa assays, were not available to ascertain blood DOAC level. Therefore, whether PCC had any DOAC level–dependent effect should be subjected to further studies. Furthermore, the median LKW-to-CT time was 3.9 hours. As hemostatic agents may confer a larger benefit among patients with ICH and onset-to-presentation within 2 hours,^35^ prospective studies with more precise time metric measurement for onset-to-presentation and last DOAC ingestion are needed to determine whether PCC treatment has a time-dependent effect on DOAC-associated ICH. Additionally, the predominant Chinese ethnicity of our cohort limits its generalizability to other ethnicities due to the interethnic variation of ICH risks.

## Conclusions

In this cohort study, Chinese patients with DOAC-associated ICH had large baseline hematoma volumes and high rates of mortality and functional disability. PCC treatment was not associated with improved functional outcome, hematoma expansion, or mortality. Further studies on novel hemostatic as well as neurosurgical and adjunctive medical therapies are needed to identify the best management algorithm of DOAC-associated ICH.

## Notes

Supplement 1. **eTable 1.** Conversion Table Between Diagnosis and ICD-9-CM Codes **eFigure.** Example of Automated Segmentation by Our nnU-Net–Based Algorithm **eTable 2.** Clinical Outcomes of the Unweighted Cohort With DOAC-Associated ICH **eTable 3.** Characteristics of the Unweighted Cohort of DOAC-Associated ICH Patients With Sequential CT Head Scans **eTable 4.** Characteristics of the Weighted Cohort of DOAC-Associated ICH Patients With Sequential CT Head Scans **eTable 5.** Characteristics of the Weighted Cohort With DOAC-Associated ICH: Atrial Fibrillation Subgroup **eTable 6.** Weighted Regression of Study Outcomes: Atrial Fibrillation Subgroup **eTable 7.** Unweighted Univariate Logistic Regression for Factors Associated With Good Neurological Outcomes **eTable 8.** Data-Driven Unweighted Multivariate Logistic Regression for Factors Associated With Good Neurological Outcomes **eTable 9.** Comparison of Patient Outcomes in the Earlier vs Later Study Period, Dichotomized by the Median Date of DOAC-Associated ICH Onset

Supplement 2.
Data Sharing Statement
