# Error in Table

**DOI:** 10.1001/jamanetworkopen.2024.5106

**Published:** 2024-02-29

In the Original Investigation titled “Prothrombin Complex Concentrate vs Conservative Management in ICH Associated With Direct Oral Anticoagulants,”^1^ published February 6, 2024, there was an error in Table 1. A row listing the number and proportion of patients receiving factor Xa inhibitors was not included. This article has been corrected.^1^
