# Supplementary material for: Prothrombin Complex Concentrate vs Conservative Management in ICH Associated With Direct Oral Anticoagulants
Source: JAMA Netw Open. 2024 Feb 6;7(2):e2354916. doi: 10.1001/jamanetworkopen.2023.54916 (PMC10848059; doi:10.1001/jamanetworkopen.2023.54916)
Supplement: Supplement 1. — eTable 1. Conversion Table Between Diagnosis and ICD-9-CM Codes eFigure. Example of Automated Segmentation by Our nnU-Net–Based Algorithm eTable 2. Clinical Outcomes of the Unweighted Cohort With DOAC-Associated ICH eTable 3. Characteristics of the Unweighted Cohort of DOAC-Associated ICH Patients With Sequential CT Head Scans eTable 4. Characteristics of the Weighted Cohort of DOAC-Associated ICH Patients With Sequential CT Head Scans eTable 5. Characteristics of the Weighted Cohort With DOAC-Associated ICH: Atrial Fibrillation Subgroup eTable 6. Weighted Regression of Study Outcomes: Atrial Fibrillation Subgroup eTable 7. Unweighted Univariate Logistic Regression for Factors Associated With Good Neurological Outcomes eTable 8. Data-Driven Unweighted Multivariate Logistic Regression for Factors Associated With Good Neurological Outcomes eTable 9. Comparison of Patient Outcomes in the Earlier vs Later Study Period, Dichotomized by the Median Date of DOAC-Associated ICH Onset [file jamanetwopen-e2354916-s001.pdf]

Supplemental Online Content

Ip B, Pan S, Yuan Z, et al. Prothrombin complex concentrate vs conservative management in ICH associated with direct oral anticoagulants. *JAMA Netw Open.* 2024;7(2):e2354916.
doi:10.1001/jamanetworkopen.2023.54916

eTable 1. Conversion Table Between Diagnosis and ICD-9-CM Codes

eFigure. Example of Automated Segmentation by Our nnU-Net–Based Algorithm

eTable 2. Clinical Outcomes of the Unweighted Cohort With DOAC-Associated ICH

eTable 3. Characteristics of the Unweighted Cohort of DOAC-Associated ICH Patients With Sequential CT Head Scans

eTable 4. Characteristics of the Weighted Cohort of DOAC-Associated ICH Patients With Sequential CT Head Scans

eTable 5. Characteristics of the Weighted Cohort With DOAC-Associated ICH: Atrial Fibrillation Subgroup

eTable 6. Weighted Regression of Study Outcomes: Atrial Fibrillation Subgroup

eTable 7. Unweighted Univariate Logistic Regression for Factors Associated With Good Neurological Outcomes

eTable 8. Data-Driven Unweighted Multivariate Logistic Regression for Factors Associated With Good Neurological Outcomes

eTable 9. Comparison of Patient Outcomes in the Earlier vs Later Study Period, Dichotomized by the Median Date of DOAC-Associated ICH Onset

This supplemental material has been provided by the authors to give readers additional information about their work.

© 2024 Ip B et al. *JAMA Network Open.*

eTable 1. Conversion Table Between Diagnosis and *ICD-9-CM* Codes

**Diagnosis**	**ICD9-CM code**
Atrial fibrillation	427.31
Congestive heart failure	428
Hypertension	401
Diabetes mellitus	250
Ischemic stroke	433, 434, 436
Intracranial hemorrhage	430, 431, 432
Ischemic heart disease	410, 411, 412, 413, 414
Venous thromboembolism	415.1, 451
Valvular replacement	42.2, 43.3

Abbreviations: ICD9-CM: *International Classification of Diseases, Ninth Revision, Clinical Modification*

© 2024 Ip B et al. *JAMA Network Open.*

eFigure. Example of Automated Segmentation by Our nnU-Net–Based Algorithm

A-F: A patients with direct oral anticoagulant-associated cerebellar hemorrhage with a haematoma volume of 2.2mL. **G-L:** The same patient with a follow-up brain computer tomography after 24 hours which noted hematoma expansion. The hematoma volume now measures 6.8mL.

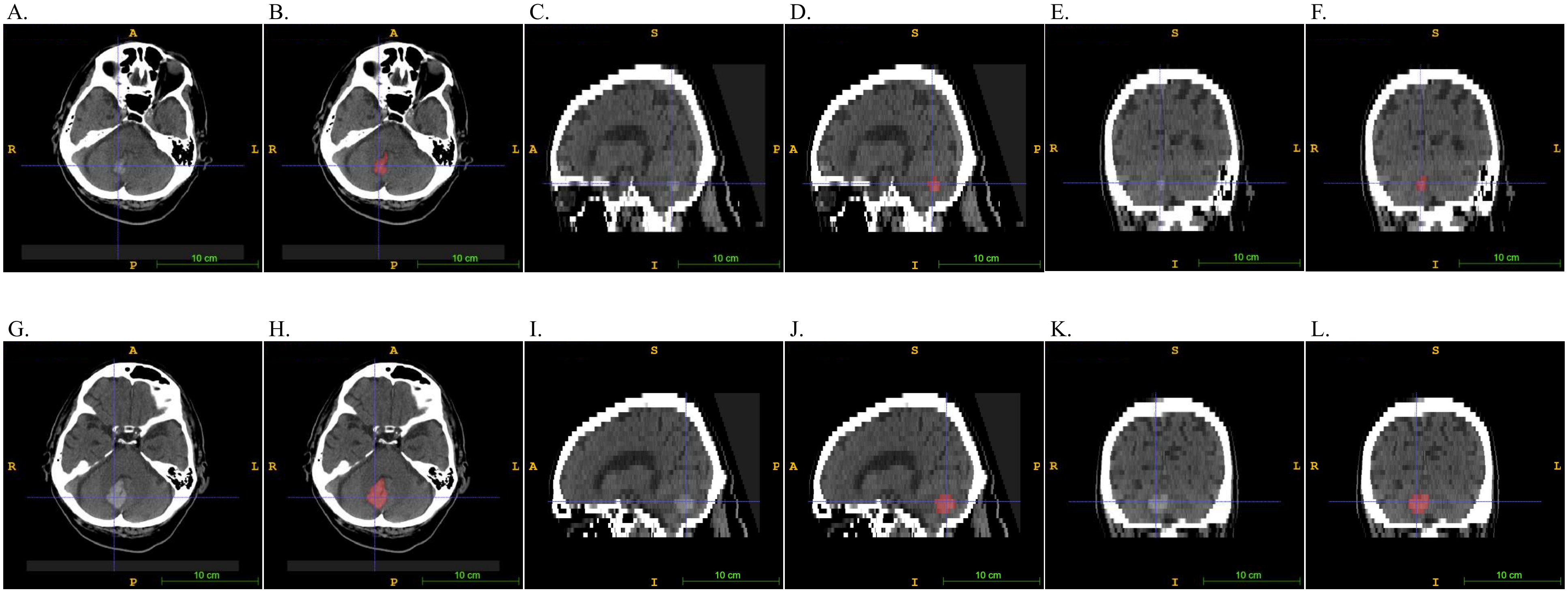

© 2024 Ip B et al. *JAMA Network Open.*

eTable 2. Clinical Outcomes of the Unweighted Cohort With DOAC-Associated ICH

	PCC (n=102)	Conservative (n=116)	Idarucizumab (n=14)
**Dosage of direct oral anticoagulants (mg), median (IQR)**			
Apixaban	5.00 (5.00-10.00)	5.00 (5.00-6.25)	/
Dabigatran	220.00 (220.00-220.00)	220.00 (220.00-220.00)	220.00 (220.00-220.00)
Edoxaban	45.00 (37.50-52.50)	45.00 (37.50-52.50)	/
Rivaroxaban	20.00 (15.00-20.00)	20.00 (15.00-20.00)	/
**Demographic**			
Age mean±sd	77.81±9.13	76.15±9.74	80.93±6.25
Female n(%)	48 (47)	48 (41)	5 (36)
Ever-smoker n(%)	33 (32)	41 (35)	7 (50)
Ever-drinker n(%)	24 (24)	34 (29)	4 (29)
**Medical co-morbidities n(%)**			
Atrial fibrillation	92 (90)	95 (82)	10 (71)
Congestive heart failure	15 (15)	11 (9)	1 (7)
Hypertension	76 (75)	78 (67)	11 (79)
Diabetes mellitus	32 (31)	34 (29)	6 (43)
Ischemic stroke	16 (16)	14 (12)	2 (14)
Ischemic heart disease	21(21)	29 (25)	3 (21)
Major bleeding	9 (9)	2 (2)	1 (7)
Venous thromboembolism	5 (5)	15 (13)	2 (14)
Intracerebral hemorrhage	0 (0)	0 (0)	
Epilepsy	7 (7)	12 (10)	0 (0)
Cancer	13 (13)	11 (9)	5 (36)
**Laboratory tests median (IQR)**			
APTT	35.05 (31.00-38.80)	34.30 (28.10-36.45)	38.35 (28.40-48.10)
PT	13.85 (12.40-16.60)	13.80 (12.40-16.12)	13.15 (12.60-13.90)
Alanine aminotransferase	19.00 (16.00-27.00)	18.00 (13.60-28.00)	15.50 (11.00-22.00)
Creatinine	89.00 (72.00-109.00)	94.00 (76.00-114.00)	92.50 (70.00-108.00)
Hb	13.25 (11.80-14.30)	12.95 (11.53-14.10)	12.10 (10.10-14.60)
Platelet	207.00 (149.00-256.00)	199.55 (159.75-257.25)	197.00 (163.00-224.00)
HbA1c	5.90 (5.70-6.60)	5.90 (5.60-6.60)	6.00 (5.80-6.90)
**Concurrent medications n(%)**			
Antiplatelet	13 (13)	22 (19)	3 (21)
Antiseizure medication	12 (12)	6 (5)	0 (0)
Nitrate	21 (21)	39 (34)	3 (21)
Calcium Channel Blocker	56 (55)	90 (78)	13 (93)
Beta Blocker	76 (75)	92 (79)	14 (100)
Statin	61 (60)	78 (67)	10 (71)
**ICH location n(%)**			
Basal ganglia	38 (37)	28 (24)	4 (29)
Thalamus	28 (27)	21 (18)	0 (0)
Lobar	37 (36)	57 (49)	10 (71)
Cerebellum/brainstem	24 (24)	23 (20)	1 (7)
**Concurrent intracranial hemorrhage n(%)**			

© 2024 Ip B et al. *JAMA Network Open.*

SDH	5 (5)	7 (6)	1 (7)
IVH	35 (34)	21 (18)	8 (57)
SAH	6 (6)	6 (5)	1 (7)
Midline shift/ Hydrocephalus/ Herniation	54 (53)	34 (29)	7 (50)
**Clinical Parameters mean±sd / median (IQR)**			
Systolic blood pressure	173.96±28.85	175.08±22.56	181.07±24.37
Diastolic blood pressure	104.12±17.68	104.15±17.55	107.14±21.09
Admission GCS	13.00 (7.00-15.00)	14.00 (10.00-15.00)	9.50 (4.00-15.00)
Premorbid mRS	2.00 (0-3.00)	1.00 (0-3.00)	2.00 (0-3.00)
Neurosurgical procedures	6 (6)	6 (5)	2 (14)
Initial hematoma volume (mean)	41.53±52.32	41.72±53.27	150.62±233.36
Initial hematoma volume	18.31 (7.23-52.21)	21.28 (2.39-58.79)	85.76 (65.92-116.81)
LKW-to-CT time (hours)	3.4 (1.3-13.1)	3.9 (1.5-15.9)	1.2 (1.2-4.8)
**Outcome n(%)**			
Good neurological outcome	24 (24)	47 (41)	1 (7)
Mortality at 90 days	44 (43)	39 (34)	7 (50)
In-hospital mortality	45 (44)	39 (34)	7 (50)

IQR: interquartile range, APTT: activated partial thromboplastin time, LKW: last-known-well, PT: prothrombin time, CT: computer tomography

SI conversion factors:

To convert creatinine to mg/dL, multiply values by 0.0113.

To convert hemoglobin to mmol/L, multiply values by 0.6206.

© 2024 Ip B et al. *JAMA Network Open.*

eTable 3. Characteristics of the Unweighted Cohort of DOAC-Associated ICH Patients With Sequential CT Head Scans

	PCC (n=60)	Conservative (n=62)
**DOAC dosage (mg), median (IQR)**		
Apixaban	5.00 (5.00-6.25)	5.00 (5.00-6.25)
Dabigatran	220.00 (220.00-220.00)	220.00 (220.00-220.00)
Edoxaban	30.00 (30.00-30.00)	30.00 (30.00-30.00)
Rivaroxaban	20.00 (17.50-20.00)	20.00 (15.00-20.00)
**Demographic**		
Age mean±sd	77.05±9.13	75.08±10.65
Female n(%)	26 (43)	28 (45)
Ever-smoker n(%)	20 (33)	21 (34)
Ever-drinker n(%)	15 (25)	19 (31)
**Medical co-morbidities n(%)**		
Atrial fibrillation	55 (92)	46 (74)
Congestive heart failure	7 (12)	5 (8)
Hypertension	44 (73)	43 (69)
Diabetes mellitus	20 (33)	19 (31)
Ischemic stroke	12 (20)	6 (10)
Ischemic heart disease	9 (15)	17 (27)
Major bleeding	4 (7)	0 (0)
Venous thromboembolism	3 (5)	13 (21)
Intracerebral hemorrhage	0 (0)	0 (0)
Epilepsy	4 (7)	9 (15)
**Laboratory tests median (IQR)**		
APTT (s)	36.15 (31.05-40.75)	34.65 (27.80-36.00)
PT (s)	14.35 (12.67-16.92)	13.40 (12.10-15.80)
Alanine aminotransferase (IU/L)	21.00 (17.00-27.00)	18.20 (13.60-29.00)
Creatinine (umol/L)	90.00 (72.00-109.00)	98.00 (68.00-114.00)
Hemoglobin (g/dL)	13.45 (11.90-14.45)	13.00 (11.60-14.10)
Platelet (10^9/L)	175.00 (145.75-239.25)	231.50 (165.00-276.00)
HbA1c (%)	6.00 (5.60-6.60)	6.00 (5.60-6.80)
**Concurrent medications n(%)**		
Antiplatelet	10 (17)	12 (19)
Antiseizure medication	6 (10)	4 (6)
Nitrate	13 (22)	24 (39)
Calcium Channel Blocker	32 (53)	50 (81)
Beta Blocker	42 (70)	51 (82)
Statin	37 (62)	44 (71)
**ICH location n(%)**		
Basal ganglia	22 (37)	14 (23)
Thalamus	18 (30)	10 (16)
Lobar	17 (28)	26 (42)
Cerebellum/brainstem	16 (27)	14 (23)

© 2024 Ip B et al. *JAMA Network Open.*

**Concurrent intracranial abnormalities n(%)**		
Subdural hemorrhage	2 (3)	4 (6)
Intraventricular hemorrhage	17 (28)	8 (13)
Subarachnoid hemorrhage	2 (3)	1 (2)
Midline shift/ Hydrocephalus/ Herniation	29 (48)	14 (23)
Neurosurgical procedures	5 (8)	3 (5)
**Clinical/radiological parameters on admission mean±sd / median (IQR)**		
Systolic blood pressure (mmHg)	171.18±27.46	170.98±21.11
Diastolic blood pressure (mmHg)	103.67±17.72	102.60±17.65
Admission Glasgow coma scale	14.50 (11.75-15.00)	15.00 (14.00-15.00)
Premorbid modified Rankin scale	1.00 (0-2.00)	1.00 (0-3.00)
LKW-to-CT time (hours)	3.2 (1.2-9.1)	3.62 (1.6-7.0)
Baseline hematoma volume (mL)	22.70±26.34	22.00±28.67
**Outcomes n(%)**		
Good neurological outcome	18 (30)	29 (47)
Mortality at 90 days	19 (32)	11 (18)
Hematoma expansion	19 (32)	17 (27)
In-hospital mortality	20 (33)	9 (15)

DOAC: direct oral anticoagulant, ICH: intracerebral hemorrhage, CT: computer tomography, last-known-well, PCC: prothrombin complex concentrate, IQR: interquartile range, APTT: activated partial thromboplastin time, PT: prothrombin time

SI conversion factors:

To convert creatinine to mg/dL, multiply values by 0.0113.

To convert hemoglobin to mmol/L, multiply values by 0.6206.

© 2024 Ip B et al. *JAMA Network Open.*

eTable 4. Characteristics of the Weighted Cohort of DOAC-Associated ICH Patients With Sequential CT Head Scans

	**PCC (n=48)**	**Conservative (n=48)**	**p-value**
**DOAC dosage (mg), median (IQR)**			
Apixaban	5.00 (5.00-10.00)	5.00 (5.00-10.00)	0.92
Dabigatran	220.00 (220.00-300.00)	220.00 (220.00-220.00)	0.17
Edoxaban	30.00 (30.00-30.00)	30.00 (30.00-60.00)	0.52
Rivaroxaban	20.00 (15.00-20.00)	20.00 (15.00-20.00)	0.97
**Demographic**			
Age mean±sd	76.33±9.64	76.53±10.12	0.93
Female n(%)	22 (46)	22 (46)	0.98
Ever-smoker n(%)	15 (30)	14 (30)	>0.99
Ever-drinker n(%)	10 (21)	13 (27)	0.49
**Medical co-morbidities n(%)**			
Atrial fibrillation	44 (90)	35 (73)	0.04
Congestive heart failure	7 (15)	4 (8)	0.33
Hypertension	35 (73)	32 (67)	0.53
Diabetes mellitus	18 (37)	15 (31)	0.54
Ischemic stroke	8 (17)	6 (12)	0.42
Ischemic heart disease	8 (17)	16 (33)	0.10
Major bleeding	3 (7)	0 (0)	0.06
Venous thromboembolism	4 (8)	9 (19)	0.19
Intracerebral hemorrhage	0 (0)	0 (0)	NA
Epilepsy	3 (7)	5 (11)	0.50
**Laboratory tests median (IQR)**			
APTT (s)	36.30 (30.90-40.70)	35.20 (29.10-39.30)	0.16
PT (s)	14.00 (12.30-16.80)	13.40 (12.20-16.00)	0.40
Alanine aminotransferase (IU/L)	22.00 (17.00-27.80)	18.00 (13.00-28.00)	0.09
Creatinine (umol/L)	91.00 (73.20-109.00)	99.00 (68.00-124.00)	0.52
Hemoglobin (g/dL)	13.30 (11.90-14.30)	13.10 (12.00-13.90)	0.48
Platelet (10^9/L)	207.00 (151.00-253.00)	233.00 (166.00-283.00)	0.15
HbA1c (%)	6.00 (5.60-6.80)	6.10 (5.70-7.10)	>0.99
**Concurrent medications n(%)**			
Antiplatelet	11 (23)	12 (24)	0.94
Antiseizure medication	4 (9)	2 (4)	0.24
Nitrate	12 (25)	19 (40)	0.12
Calcium Channel Blocker	26 (53)	39 (81)	0.002
Beta Blocker	34 (70)	38 (79)	0.37
Statin	31 (64)	36 (74)	0.30
**ICH location n(%)**			
Basal ganglia	16 (33)	14 (30)	0.77
Thalamus	12 (25)	8 (17)	0.30
Lobar	17 (35)	16 (34)	0.92
Cerebellum/brainstem	12 (25)	12 (25)	0.98

© 2024 Ip B et al. *JAMA Network Open.*

**Concurrent intracranial abnormalities n(%)**			
Subdural hemorrhage	1 (2)	2 (5)	0.38
Intraventricular hemorrhage	10 (20)	9 (18)	0.82
Subarachnoid hemorrhage	2 (4)	3 (6)	0.74
Midline shift/ Hydrocephalus/ Herniation	17 (35)	14 (30)	0.61
**Clinical/radiological parameters on admission mean±sd / median (IQR)**			
Systolic blood pressure (mmHg)	170.98±25.75	170.33±21.06	0.89
Diastolic blood pressure (mmHg)	103.32±17.33	101.56±18.51	0.63
Admission Glasgow coma scale	15.00 (12.00-15.00)	15.00 (14.00-15.00)	0.81
Premorbid modified Rankin scale	1.00 (0-2.00)	1.00 (0-3.00)	0.78
LKW-to-CT time (hours)	3.2 (1.2-9.1)	3.62 (1.6-7.0)	0.332
Baseline hematoma volume (mL)	22.16±29.71	21.47±28.69	0.910
Neurosurgical procedures	4 (8)	3 (7)	0.87
**Outcomes n(%)**			
Good neurological outcome	15 (31)	21 (43)	0.25
Mortality at 90 days	14 (28)	9 (19)	0.26
Hematoma expansion	13 (27)	13 (27)	0.97
In-hospital mortality	13 (27)	8 (17)	0.22

DOAC: direct oral anticoagulant, ICH: intracerebral hemorrhage, CT: computer tomography, last-known-well, PCC: prothrombin complex concentrate, IQR: interquartile range, APTT: activated partial thromboplastin time, PT: prothrombin time

SI conversion factors:

To convert creatinine to mg/dL, multiply values by 0.0113.

To convert hemoglobin to mmol/L, multiply values by 0.6206.

© 2024 Ip B et al. *JAMA Network Open.*

eTable 5. Characteristics of the Weighted Cohort With DOAC-Associated ICH: Atrial Fibrillation Subgroup

	PCC (n=76)	Conservative (n=62)	p-value
**DOAC dosage (mg), median (IQR)**			
Apixaban	5.00 (5.00-10.00)	5.00 (5.00-5.00)	0.44
Dabigatran	220.00 (220.00-220.00)	220.00 (150.00-220.00)	0.09
Edoxaban	30.00 (30.00-60.00)	60.00 (60.00-60.00)	0.33
Rivaroxaban	20.00 (15.00-20.00)	20.00 (15.00-20.00)	0.83
**Demographic**			
Age mean±sd	77.59±9.01	77.28±8.92	0.84
Female n(%)	37 (49)	32 (51)	0.76
Ever-smoker n(%)	23 (30)	17 (28)	0.80
Ever-drinker n(%)	17 (23)	15 (24)	0.88
**Medical co-morbidities n(%)**			
Congestive heart failure	13 (17)	4 (7)	0.04
Hypertension	59 (78)	47 (76)	0.78
Diabetes mellitus	27 (35)	22 (35)	0.99
Ischemic stroke	11 (14)	10 (17)	0.73
Ischemic heart disease	14 (19)	14 (23)	0.52
Major bleeding	9 (12)	2 (2)	0.05
Venous thromboembolism	4 (5)	3 (5)	0.96
Intracerebral hemorrhage	0 (0)	0 (0)	NA
Epilepsy	5 (7)	4 (7)	0.97
**Laboratory tests median (IQR)**			
APTT (s)	35.10 (30.20-38.20)	33.70 (28.80-39.30)	0.58
PT (s)	13.80 (12.10-16.60)	13.50 (12.30-16.50)	0.70
Alanine aminotransferase (IU/L)	18.00 (15.10-27.00)	17.00 (14.00-27.00)	0.42
Creatinine (umol/L)	88.00 (72.00-105.00)	98.00 (79.00-114.00)	0.08
Hemoglobin (g/dL)	13.20 (11.70-14.20)	13.10 (11.70-14.10)	0.71
Platelet (10^9/L)	210.00 (145.00-248.00)	196.00 (157.00-271.00)	0.83
HbA1c (%)	5.90 (5.70-6.70)	6.00 (5.70-6.60)	0.86
**Concurrent medications n(%)**			
Antiplatelet	12 (16)	9 (15)	0.80
Antiseizure medication	7 (9)	2 (4)	0.18
Nitrate	14 (19)	24 (39)	0.01
Calcium Channel Blocker	46 (60)	47 (76)	0.04
Beta Blocker	55 (72)	49 (80)	0.29
Statin	46 (60)	44 (72)	0.10
**ICH location n(%)**			
Basal ganglia	27 (36)	19 (31)	0.61
Thalamus	18 (24)	15 (24)	0.95
Lobar	32 (42)	23 (38)	0.62
Cerebellum/brainstem	17 (23)	12 (20)	0.74
**Concurrent intracranial abnormalities n(%)**			
Subdural hemorrhage	3 (4)	3 (5)	0.61
Intraventricular hemorrhage	21 (27)	19 (31)	0.60
Subarachnoid hemorrhage	4 (5)	2 (4)	0.78

© 2024 Ip B et al. *JAMA Network Open.*

Midline shift/ Hydrocephalus/ Herniation	33 (43)	24 (39)	0.66
**Clinical/radiological parameters on admission mean±sd / median (IQR)**			
Systolic blood pressure (mmHg)	175.61±26.40	175.62±23.98	>0.99
Diastolic blood pressure (mmHg)	105.00±17.07	104.60±18.49	0.89
Admission Glasgow coma scale	14.00 (9.00-15.00)	14.00 (9.00-15.00)	0.82
Premorbid modified Rankin scale	2.00 (0-2.00)	1.00 (0-3.00)	0.83
LKW-to-CT time (hours)	3.4 (1.3-13.1)	3.9 (1.5-15.9)	0.22
Baseline hematoma volume (mL)	17.49 (4.19-44.65)	16.45 (2.60-61.12)	0.66
Neurosurgical procedures n(%)	3 (4)	2 (4)	>0.99
**Outcomes n(%)**			
Good neurological outcome	20 (26)	20 (33)	0.37
Mortality at 90 days	29 (38)	23 (37)	0.90
In-hospital mortality	28 (37)	24 (39)	0.79

DOAC: direct oral anticoagulant, ICH: intracerebral hemorrhage, last-known-well, PCC: prothrombin complex concentrate, IQR: interquartile range, APTT: activated partial thromboplastin time, PT: prothrombin time, CT: computer tomography

SI conversion factors:

To convert creatinine to mg/dL, multiply values by 0.0113.

To convert hemoglobin to mmol/L, multiply values by 0.6206.

© 2024 Ip B et al. *JAMA Network Open.*

eTable 6. Weighted Regression of Study Outcomes: Atrial Fibrillation Subgroup

	Prothrombin complex concentrate (n=76) vs Conservative management (Referent, n=62)	
**Outcomes**	**aOR (95% CI)**	**p-value**
Good neurological recovery at three months	0.72 (0.35-1.48)	0.37
Mortality at three months	0.99 (0.61-1.61)	0.98
In-hospital mortality	1.01 (0.58-1.75)	0.98
Hematoma expansion[1]	0.73 (0.25-2.11)	0.55

aOR: adjusted odds ratio, CI: confidence interval

[1] 48 patients in the prothrombin complex concentrate group and 47 patients in the conservative management group had sequential brain computer tomography for comparison of hematoma expansion

© 2024 Ip B et al. *JAMA Network Open.*

eTable 7. Unweighted Univariate Logistic Regression for Factors Associated With Good Neurological Outcomes

	Univariable model (p < 0.1)	
Covariates	OR (95% CI)	p-value
Baseline hematoma volume	0.96 (0.94-0.97)	<0.001
Intraventricular hemorrhage	0.13 (0.05-0.34)	<0.001
Glasgow coma scale on admission	1.42 (1.24-1.62)	<0.001
Premorbid mRS	0.84 (0.70-1.01)	0.06
Subarachnoid hemorrhage	0.17 (0.02-1.38)	0.10
Midline shift/ Hydrocephalus/ Herniation	0.33 (0.18-0.63)	0.001
Prothrombin complex concentrate	0.55 (0.31-0.99)	0.05
Congestive heart failure	0.35 (0.12-1.06)	0.06

OR: odds ratio, CI: confidence interval, mRS: modified Rankin Scale

eTable 8. Data-Driven Unweighted Multivariate Logistic Regression for Factors Associated With Good Neurological Outcomes

	Multivariate model	
**Covariates**	**aOR (95% CI)**	**p-value**
Baseline hematoma volume	0.97 (0.95-0.99)	0.001
Intraventricular hemorrhage	0.29 (0.09-0.88)	0.03
Glasgow coma scale on admission	1.21 (1.04-1.41)	0.02
Premorbid mRS	0.90 (0.70-1.16)	0.41
Subarachnoid hemorrhage	0.80 (0.07-9.08)	0.86
Midline shift/ Hydrocephalus/ Herniation	0.96 (0.42-2.22)	0.93
Prothrombin complex concentrate	0.58 (0.28-1.22)	0.15
Congestive heart failure	0.48 (0.13-1.81)	0.28

aOR: adjusted odds ratio, CI: confidence interval, modified Rankin Scale

© 2024 Ip B et al. *JAMA Network Open.*

eTable 9. Comparison of Patient Outcomes in the Earlier vs Later Study Period, Dichotomized by the Median Date[1] of DOAC-Associated ICH Onset

	Earlier than median date (n = 116)	Later than median date (n = 116)	p-value
Mortality at 90 days	71 (61%)	60 (52%)	0.19
In-hospital mortality	39 (34%)	52 (45%)	0.11
Good neurological outcome	32 (28%)	29 (25%)	0.77
Hematoma expansion[2]	20 (31%)	20 (30%)	>0.99

DOAC: direct oral anticoagulant, ICH: intracerebral hemorrhage

[1]The study period was 1st January 2016 to 31st December 2021, while the median date of DOAC-associated ICH onset was 1st June 2018

[2]A total of 130 patients had sequential brain computer tomography for the comparison of hematoma expansion

© 2024 Ip B et al. *JAMA Network Open.*
