# Supplementary material for: Prothrombin Complex Concentrate vs Conservative Management in ICH Associated With Direct Oral Anticoagulants
Source: JAMA Netw Open. 2024 Feb 6;7(2):e2354916. doi: 10.1001/jamanetworkopen.2023.54916 (PMC10848059; doi:10.1001/jamanetworkopen.2023.54916)
Supplement: Supplement 2. — Data Sharing Statement [file jamanetwopen-e2354916-s002.pdf]

Data Sharing Statement

Ip. Prothrombin Complex Concentrate vs Conservative Management in ICH Associated With Direct Oral Anticoagulants. *JAMA Netw Open*. Published February 06, 2024.
doi:10.1001/jamanetworkopen.2023.54916

Data
Data available: No

Additional Information
Explanation for why data not available: Due to the data and privacy protection policy of the Hospital Authority Data Collaboration Laboratory, individual patient data cannot be shared. Anonymized individual patient data from 70 patients in the direct oral anticoagulant registry will be made availabile on request from qualified investigators for research purpose.
